# Bovine Colostrum Increases Pore-Forming Claudin-2 Protein Expression but Paradoxically Not Ion Permeability Possibly by a Change of the Intestinal Cytokine Milieu

**DOI:** 10.1371/journal.pone.0064210

**Published:** 2013-05-23

**Authors:** Peggy Bodammer, Claus Kerkhoff, Claudia Maletzki, Georg Lamprecht

**Affiliations:** 1 Fraunhofer Institute for Cell Therapy and Immunology, Department of Immunology, Project group “Extracorporal Immunomodulation”, Rostock, Germany; 2 Division of Gastroenterology, Department of Medicine II, University of Rostock, Rostock, Germany; 3 Department of General, Vascular, Thoracic and Transplantation Surgery, Section of Molecular Oncology and Immunotherapy, University of Rostock, Rostock, Germany; University of Chicago, United States of America

## Abstract

An impaired intestinal barrier function is involved in the pathogenesis of inflammatory bowel disease (IBD). Several nutritional factors are supposed to be effective in IBD treatment but scientific data about the effects on the intestinal integrity remain scarce. Bovine colostrum was shown to exert beneficial effects in DSS-induced murine colitis, and the present study was undertaken to explore the underlying molecular mechanisms. Western blot revealed increased claudin-2 expression in the distal ileum of healthy mice after feeding with colostrum for 14 days, whereas other tight junction proteins (claudin-3, 4, 10, 15) remained unchanged. The colostrum-induced claudin-2 induction was confirmed in differentiated Caco-2 cells after culture with colostrum for 48 h. Paradoxically, the elevation of claudin-2, which forms a cation-selective pore, was neither accompanied by increased ion permeability nor impaired barrier function. In an *in situ* perfusion model, 1 h exposure of the colonic mucosa to colostrum induced significantly increased mRNA levels of barrier-strengthening cytokine transforming growth factor-β, while interleukine-2, interleukine-6, interleukine-10, interleukine-13, and tumor-necrosis factor-α remained unchanged. Thus, modulation of the intestinal transforming growth factor-β expression might have compensated the claudin-2 increase and contributed to the observed barrier strengthening effects of colostrum *in vivo and in vitro*.

## Introduction

The gastrointestinal epithelium comprised a monolayer of epithelial cells that forms a physical barrier to the external environment. It displays a number of specialized protective functions to prevent direct access of microorganisms and antigens to the intestinal mucosa [1]. The epithelial monolayer facilitates the transcellular and the paracellular transport. [2]. The tight junction (TJ) provides both a barrier to luminal molecules, including bacterial antigens as well as food components, and a pore for ions and solutes [3], [4].

The gastrointestinal immune system faces a unique challenge to maintain homeostasis between the resident microbiota and the host tissue and to mount a controlled inflammatory response to pathogens [5]. In inflammatory bowel diseases (IBD) the loss of tolerance to the normal microbiota is one trigger that causes mucosal damage. The deregulated communication between the intestinal microbiota and the mucosal immune system in the genetically predisposed host is the common paradigm for the pathogenesis of IBD [6]. In addition, environmental factors including diet and food additives may be etiological factors in IBD [7].

Crohn’s disease and ulcerative colitis are chronic inflammatory disorders of the gut that cause major life-long disability. IBD represents an important public health problem affecting the patients’ education, working abilities, social life and quality of life. IBD cannot be cured. The goal of current therapies is to control the clinical signs of inflammation using various pharmacologic approaches. A large number of nutritional factors are supposed to exert anti-inflammatory properties in the context of gastrointestinal disorders such as IBD. Such factors may have either a preventive or prophylactic effect against IBD or their lack of in the diet may even have a role in its pathogenesis. Therefore, there is growing interest in the immunomodulatory and barrier enhancing effects of dietary supplements and food components [8], [9]. Therapies based on complementary alternative medical strategies are widely-used by patients with IBD in addition to conventional drugs. They include traditional practices e. g. homeopathy and chinese medicine as well as the use of naturally occurring substances such as herbals and bovine colostrum (BC) [10].

Colostrum is produced by mammals during the first three days post-partum. It has a range of functional constituents including immunoglobulins, growth factors, and antimicrobial peptides [11]. The potency of BC to improve infectious and inflammatory gastrointestinal diseases is well established [12]–[15]. However, the underlying mechanisms beyond anti-inflammatory effects are not well understood. In our previous study, we observed beneficial effects of prophylactic BC application in dextran sodium sulfate-induced murine colitis. BC promoted recovery from epithelial damage by the induction of immunoregulatory cells [16].

The epithelial barrier function is partly regulated by the expression of TJ proteins and functional TJ abnormalities are associated with the pathogenesis of Crohn’s disease and ulcerative colitis [17], [18]. Claudin-2 was the first described channel-forming TJ protein facilitating cation permeability [19] and paracellular water transport [20]. Although claudin-2 is the most extensively studied charge-selective claudin channel, its functional role in health and gastrointestinal disease remains unclear. Claudin-2 is physiologically expressed in leaky epithelia such as the proximal kidney tubule and the intestine [21]. Its expression is up-regulated in epithelial cells under pro-inflammatory conditions [22]–[25]. In the presence of pro-inflammatory cytokines, such as interleukine (IL)-6, IL13, and tumor necrosis factor (TNF)-α, claudin-2 mediates elevated cation- and water flux, resulting in lower transepithelial electrical resistance (TER) [19], [20], [26]. Increased claudin-2 levels can also be found in the mucosa of patients with active IBD [18], [22]–[24], [27]. However, little is known about the functional expression of claudin-2 in healthy mucosal tissue.

The present study aimed to unravel the underlying mechanisms of BC that lead to enhanced barrier function in the context of acute mucosal injury. We found that BC increased exclusively claudin-2 protein expression in both murine healthy intestine and in an epithelial cell line. Although claudin-2 is known to increase ion permeability, its up-regulated expression was not accompanied by a lower TER. *In situ* colon perfusion experiments revealed that short-time BC-exposure influences the local cytokine milieu by increased expression of the barrier strengthening cytokine transforming growth factor-β (TGF-β). An immunomodulatory effect may therefore have contributed to the observed beneficial effects in experimental colitis.

## Materials and Methods

### Animal Studies

All animal experiments were performed according to the German protection law and were approved by the Committee on the Ethics of Animal Experiments of the University of Rostock (Landesamt für Landwirtschaft, Lebensmittelsicherheit und Fischerei Mecklenburg-Vorpommern, Permit Number: LALLF M-V/TSD/7221.3-1.1-059/08).

Female outbred NMRI mice aged eight to twelve months (average body weight 30 g) were purchased from Harlan Winkelmann GmbH (Melderslo, Netherlands). They received standard food and water *ad libitum*. For the animal studies, bovine colostrum powder (SANIMALIS, Heinsberg, Germany) was skimmed, pasteurized, and freeze-dried to ensure minimum denaturation of immunoglobulins and nutrients. The total protein content of the colostrum preparation was 60–70 g/100 g. The concentrations of immunoglobulins and other major ingredients are indicated in Table 1. For oral application, BC powder was dissolved in water. Mice received a total volume of 100 µl daily containing a dose of 20 mg freeze-dried BC per kg body weight by oral gavage for 14 days (BC, n = 6). Animals with no intervention served as controls (untreated, n = 6).

**Table 1:** Composition of bovine colostrum (SANIMALIS, Heinsberg, Germany).

Nutritional facts	in [g/100 g]
**Total fat**	<2
**Carbohydrates**	15–20
**Total proteine**	60–70
**Immunoglobulins**	
IgG	25
IgA	1.5–3.5
IgM	0.5–1.0
**Antimicrobial peptide**	
Lactoferrin	∼ 0.1
**Further ingredients**	
Ashes	6.0–8.5
Lactose	15–20

For single-pass perfusion experiments mice were fastened over-night and anesthetized with ketamine (100 mg/kg body weight, intraperitoneal) and xylazine (15 mg/kg body weight, intraperitoneal). Anesthesia was maintained by using 50% of the initial dose ketamine solution every 30–45 min. Mice were placed on a heat-plate (37°C) and the abdomen was opened by a small incision along the linea alba. The colon was identified and gently flushed with a pre-heated (37°C) sodium chloride solution. Then the proximal end of the colon near the cecum was ligated with intact blood supply, isolated by a small incision, cannulated with a silicone tube (Portex® ID: 2 mm; OD: 3 mm) and secured. Another silicone tube was advanced to the rectum to allow distal drainage and the abdomen was closed. The colon was perfused (Varioperpex® II pump) at a flow rate of 5 ml/h. After an initial 10-min washout with 0.9% sodium chloride solution, 5% BC diluted in Hank’s balanced salt solution (HBSS) or HBSS alone (control) was perfused single pass for 1 h. The colon was removed and the length was measured. Proximal tissue biopsies were frozen (−80°C) for subsequent RNA-isolation and real-time PCR cytokine expression analysis (IL-2, IL-6, IL-10, IL-13, TGF-β, TNF-α).

### RNA-isolation and Real-time PCR

Total RNA from proximal colon biopsies (20–30 mg) was isolated with TRIzol® reagent according to the manufacturer’s instructions. RNA was reverse transcribed into cDNA from 0.25 µg RNA using the High Capacity cDNA Reverse Transcription Kit (Applied Biosystems, USA). Target cDNA levels were analyzed by quantitative real-time PCR using TaqMan™Universal PCR Master Mix and pre-designed TaqMan® gene expression assays (Mm00446190_m1 (IL-6), Mm00439616_m1 (IL-10), Mm 00434204_m1 (IL-13), Mm00434256_m1 (IL-2), Mm01178820_m1 (TGF-β), Mm00443258_m1 (TNF-α), Mm99999915_g1 (GAPDH, housekeeping gene control) in an ABI Prism 7000 sequence detection system (Applied Biosystems, USA). PCR conditions were as follows: 95°C for 10 min, 55 cycles of 15 s at 95°C, 1 min at 60°C. Reactions were performed in triplicates. The expression levels of the gene of interest are given in relation to the housekeeping gene (_Δ_Ct = Ct_target_-Ct_GAPDH_). Relative gene expression values are expressed as 2^−(ΔCt)^.

### Cell Culture

Caco-2 cells (ACC169) were obtained from the German Collection of Microorganisms and Cell Cultures (DSMZ, Leibnitz-Institute, Braunschweig, Germany). Cells were maintained in complete Dulbecco’s Modified Eagle Medium/Ham’s F12 containing stable glutamine (Biochrom, Germany), 10% fetal bovine serum (FBS, PAA, Germany) and 1% penicillin/streptomycin (PAA, Germany) in a humidified atmosphere by 37°C and 5% CO_2_. For in vitro studies cell passages between 9 and 43 were used.

Soluble, sterile BC (SANIMALIS, Germany) was diluted in complete cell culture media to a final concentration of 5% BC. FBS-enriched complete media, containing additional 5% FBS, was provided as control to exclude unspecific effects of BC.

### Preparation of Whole Cell Extracts from Epithelial Cells and Tissue Samples

Caco-2 cells were seeded in 6-well plates (0.5×10^6^ per well) and grown to confluence under standard conditions. BC was applied to the cells for 48 h. Since IL-6 is known to induce claudin-2 expression [22], human recombinant IL-6 (50 ng/ml, Immunotools, Germany) was applied for additional 24 h. Cell supernatants were collected for determining IL-8 cytokine secretion using conventional ELISA technique (Fisher Scientific, Germany). For total cellular protein extraction, monolayers were gently washed with phosphate buffered saline and cells were lysed by adding 500 µl sample buffer directly (4× concentrated; 1 M Tris, pH 6.9, 8% (w:v) sodium dodecyl sulphate, 40% (v:v) glycerol, 1.5% (w:v) dithiothreitol, 1% (w:v) bromphenolblue) to the monolayer. Cell extracts were heated by 100°C for 5 min.

To obtain tissue protein extracts, distal ileal biopsies (30 mg) from mice treated with BC and untreated controls were homogenized on liquid nitrogen and incubated with lysis buffer (1 M Tris, pH 7.5, 5 M sodium chloride, 0.25 M ethylenediaminetetraacetic acid, 10% (v:v) triton-x 100, 4% (v:v) sodium azide, 0.1 M phenylmethanesulfonylfluoride, protease inhibitor cocktail (Roche, Germany) on ice for 30 min. Cell lysates were centrifuged by 10,000×g for 10 min and 4°C. Protein concentration was determined using Bradford reagent (Bio-Rad, Germany) according to manufacture’s instructions.

### Immunoblot Analysis

Proteins (20 µg) were separated by 12% SDS-polyacrylamide gel electrophoresis and transferred to polyvinylidene fluoride membranes. Membranes were blocked for 1 h with blocking buffer (Rockland-Inc., United States) prior to incubation over night with specific primary antibodies for claudin-2, claudin-3, claudin-4, claudin-10, claudin-15 (all Zymed®, Invitrogen, Germany) and β-actin (New England Biolabs, Germany). IRDye® 800 CW and IRDye® 680 CW- conjugated secondary antibodies (LI-COR-Biosciences, Germany) were applied for 30 min. Immunoblots were scanned at a wavelength of 700 nm for detecting IRDye® 680 labeled antibodies and at a wavelength of 800 nm for IRDye® 800 CW conjugated antibodies using an Odyssey® Infrared Imaging System (LI-COR-Biosciences, Germany). Signal integrated intensities were quantified using the Odyssey® software version 3.16. Probes were normalized by calculating the ratio of the corresponding claudin-protein to the β-actin signal.

### Immunfluorescence

Caco-2 cells were seeded on plastic cover slips in 24-well plates (0.1×10^6^ per well of a 24- well plate) and grown to confluence under standard conditions. Analogous to immunoblot analysis, BC was added to 14 days confluent differentiated cells for 48 h. Cell monolayers were gently washed with phosphate buffered saline, fixed with ice-cold methanol for 10 min and blocked with 2% (w:v) bovine serum albumin (BSA, Sigma) for 1 h prior to incubation with rabbit polyclonal anti-claudin-2 primary antibody for 2 h. Cells were subsequently incubated with Alexa 546 goat anti-mouse IgG (Invitrogen, Germany) for 30 min and nuclei were counterstained with 4′,6-diamidino-2-phenylindole. The specimens were embedded in mounting-medium and fluorescence imaging was performed with Olympus FV10i confocal microscope (Olympus, Germany) using FV10-ASW 3.1 software.

### Measurement of Transepithelial Electrical Resistance

In order to analyze TJ permeability to ionic solutes, TER was recorded in filter-grown differentiated Caco-2 cells as a measure of ion permeability. Cells were grown for at least 14 days after confluence, or until monolayers reached TER values of 300 Ω * cm^2^ on cell culture inserts (6.4 mm, 0.3 cm^2^, BD Falcon™, Germany) in 24 well plates. BC was diluted in complete cell culture media to a 5% solution and added to the apical aspect of the cells. FBS-enriched complete media, containing additional 5% FBS, served as a control for specific effects of BC. Since IL-6 is known to reduce TER [22], human recombinant IL-6 (50 ng/ml, Immunotools, Germany) was applied to the apical side of some wells (positive control). TER was measured using a Millicell-ERS Electrical Resistence System (Merck Millipore, Germany) at varying time points up to 72 h.

### Measurement of the Permeability for Uncharged Macromolecules

For studying TJ permeability to uncharged macromolecules, differentiated Caco-2 cells obtained 14 days post confluent (TER at least 300 Ω * cm^2^) were grown on cell culture inserts (6.4 mm, 0.3 cm^2^, BD Falcon™, Germany) in 24 well plates. After 48 h culture with 5% BC or 5% FBS-enriched control media, 4 kDa fluorescein isothiocyanate-dextrane (4 kDa FITC-Dx; 2 mg/ml, Sigma, Germany) was applied to the apical well and cells were incubated for another 4 h. Unidirectional flux of 4 kDa FITC-Dx (excitation 495 nm, emission: 525) was measured in the basal well using a fluorescence plate reader (Tecan i control infinite 200, Germany).

### Statistics

Values are reported as mean ± standard error of the mean (SEM). Group comparisons were performed by U-test using SPSS software. P<0.05 was considered to be statistically significant.

## Results

### BC Induces Claudin-2 Expression in the Distal Ileum of Healthy Mice

The permeability of the mucosa barrier mainly depends on the dynamic assembly of TJ proteins. Since BC-feeding promoted epithelial recovery, we analyzed TJ-expression in non-inflamed ileal tissue of control mice after 14 days of BC-treatment. BC-fed mice did not show any signs of inflammation such as weight loss, occult blood, diarrhea or histological damage (data not shown).

**Figure 1:**
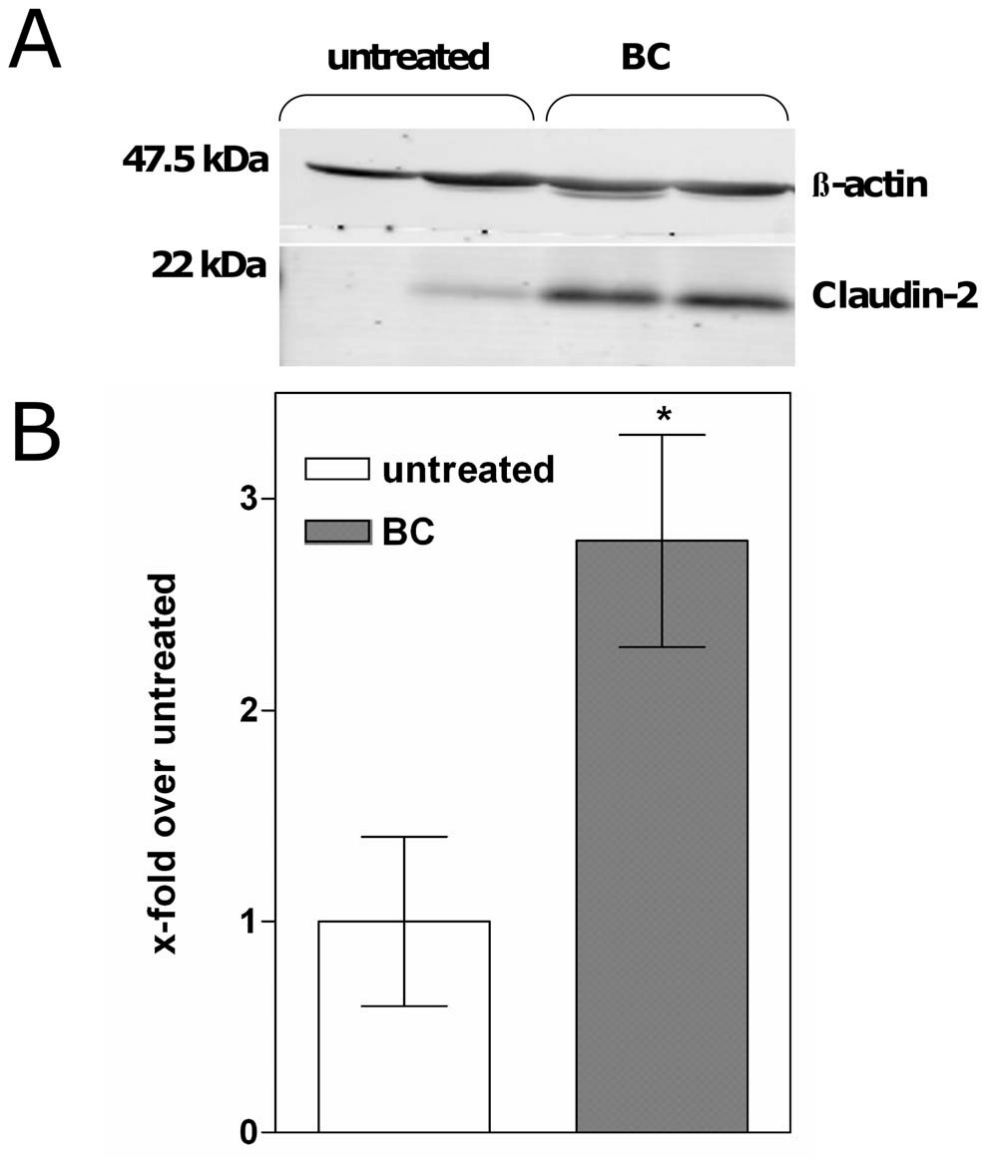
Bovine colostrum increases claudin-2 expression in the distal ileum of healthy mice. (A) Representative western blot of elevated claudin-2 protein expression in whole tissue extracts from the distal ileum of mice after 14 days feeding with bovine colostrum (BC) and untreated controls. β-actin was used as internal control for equal protein loading. (B) Increased relative expression of claudin-2 protein upon BC-feeding compared to untreated controls. *p<0.05 vs. untreated, n = 5–6.

By western blot claudin-2 expression was 2-fold increased in the distal ileum of BC-fed mice compared to untreated controls (1.0±0.4 vs. 2.8±0.5, p = 0.03) (Fig. 1A and B). Of note, the expression of other sealing (claudin-4, claudin-3) or pore-forming claudins (claudin-10, claudin-15) remained unaffected (data not shown).

### BC Increases Claudin-2 Expression in Epithelial Cells

**Figure 2:**
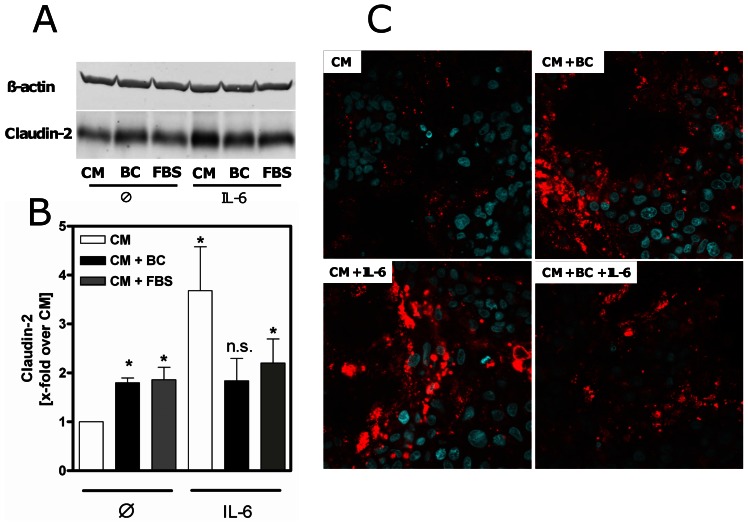
Changes of claudin-2 protein expression in intestinal epithelial cells. (A) Western blot analysis revealed elevated claudin-2 expression in 14 days postconfluent differentiated Caco-2 cells after 48 h incubation with bovine colostrum (BC) or fetal bovine serum (FBS) compared to complete media (CM). β-actin was used as internal control for equal protein loading. (B) Relative expression of claudin-2 protein in Caco-2 cells was increased upon 48 h pre-incubation with BC compared to CM and after stimulation with IL-6 (50 ng/ml) for 24 h. *p<0.05 vs. CM, n = 5–6. (C) Immunofluorescence of Caco-2 cells after incubation with BC confirmed elevation of claudin-2 (red) by BC and IL-6 (24 h, 50 ng/ml) compared to CM after 48 h. Nuclei were counterstained with 4′,6-diamidino-2-phenylindole (blue). Representative images of 3 individual experiments. Magnification: ×600.

The observed effect on claudin-2 protein expression was confirmed in differentiated Caco-2 cells after treatment with BC for 48 h. BC significantly induced claudin-2 protein levels in these cells (1.8 fold ±0.1, p<0.002, vs. CM). Of note, incubation with FBS also increased claudin-2 expression (1.8-fold ±0.3, p<0.004, vs. CM). Since claudin-2 expression is induced by IL-6, we used this cytokine as a positive control. As expected, IL-6 strongly induced claudin-2 (3.7-fold ±0.9, p = 0.004, vs. CM) protein expression. Noteworthy, pre-incubation with BC had no additive effects on IL6-induced claudin-2 expression (Fig. 2A and B).

Immunofluorescence of BC-exposed Caco-2 cells further confirmed the effect of BC on claudin-2 protein expression. BC-induced claudin-2 expression was comparable with the effect observed by IL-6. In addition, there was a lower response for the stimulation of BC-pre-incubated cells to IL-6 (Fig. 2C).

### Elevated Claudin-2 Expression is not Paralleled by Decreased Transepithelial Electrical Resistance

Since claudin-2 is a pore-forming TJ protein, mediating specific paracellular permeability for small cations, we measured the TER of differentiated filter-grown Caco-2 cells after BC exposure. Increased ion permeability should be reflected in declined TER values.

**Figure 3:**
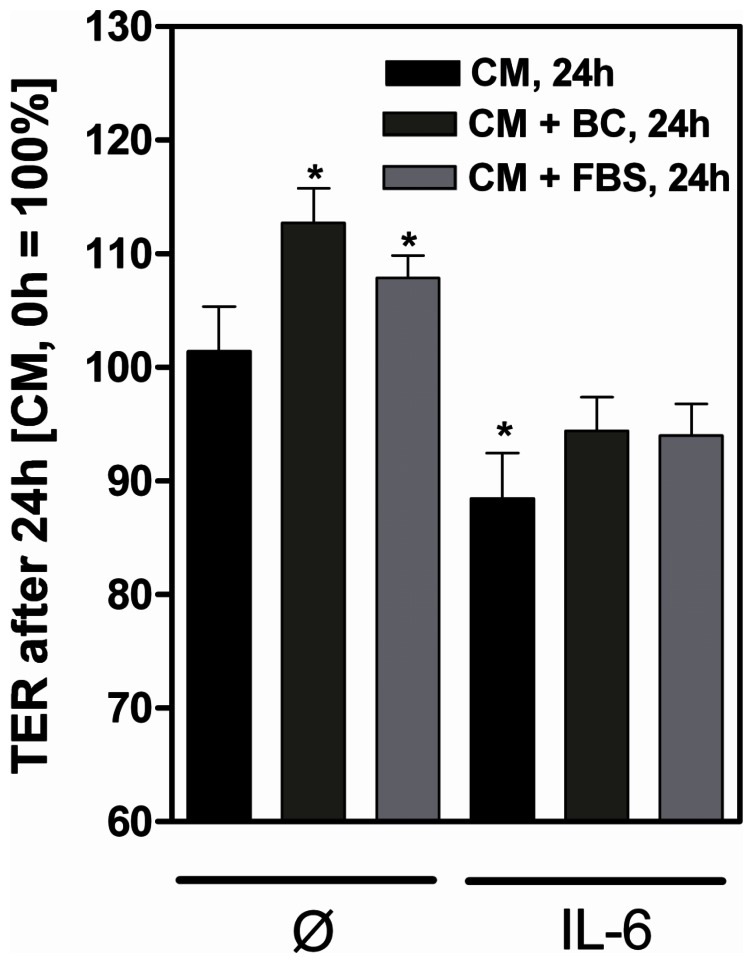
Increased claudin-2 expression is not paralled by decreased transepithelial electrical resistance. Apical stimulation of filter-grown differentiated 14 days postconfluent Caco-2 cells with IL-6 (50 ng/ml) decreased transepithelial electrical resistance (TER) after 24 h compared to unstimulated controls (complete media, CM). Incubation with bovine colostrum (BC) or fetal bovine serum (FBS) increased TER. TER in the presence of CM at the beginning of the incubation was set as 100%. *p<0.05, n = 3–4.

As expected, stimulation with IL-6 significantly reduced the TER of differentiated Caco-2 cells after 24 h (88.5% +4.0%, CM+IL-6, 24 h vs. CM, 0 h, 24 h, p = 0.02). Unexpectedly, although BC and FBS induced claudin-2 expression similar to IL-6, stimulation of cells with BC or FBS was not accompanied by decreased TER. Contrary, TER values were significant elevated upon 24 h stimulation with both substances (p = 0.006 vs. BC, p = 0.002 vs. FBS) (Fig. 3).

### BC does not Impair the Integrity of Epithelial Cells

**Figure 4:**
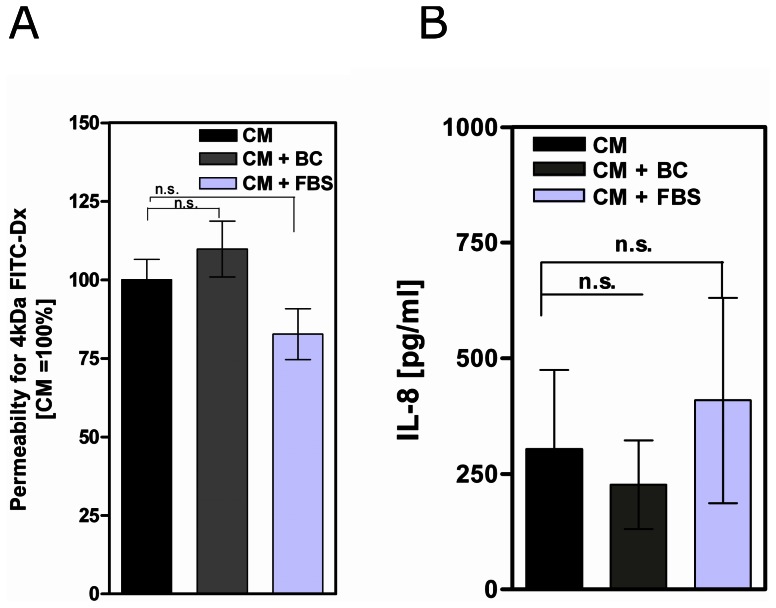
Bovine colostrum does not impair integrity of Caco-2 monolayers. (A) Permeability of filter-grown differentiated 14 days postconfluent Caco-2 cells for 4 kDa fluorescein isothiocyanate-dextrane and IL-8 cytokine secretion (B) were not affected by 48 h incubation with bovine colostrum (BC) or fetal bovine serum (FBS) compared to complete media (CM, control), n = 4 per assay and per group.

Since claudin-2 is up-regulated under inflammatory conditions, we analyzed whether or not BC influences the integrity of differentiated filter-grown epithelial cells. Therefore, permeability of Caco-2 monolayers for uncharged macromolecules was measured by 4 kDa FITC-Dx assay and IL-8 cytokine secretion was assayed as a marker for epithelial cell damage. As shown in Fig. 4, neither BC nor FBS-treated cells show significant different permeability for 4 kDa FITC-Dx (Fig. 4A), and did not provoke IL-8 secretion compared to controls (CM) after 48 h (Fig. 4B). Thus, BC did not induce detectable damage to the Caco-2 monolayer.

### BC Influences the Intestinal Cytokine Milieu

**Figure 5:**
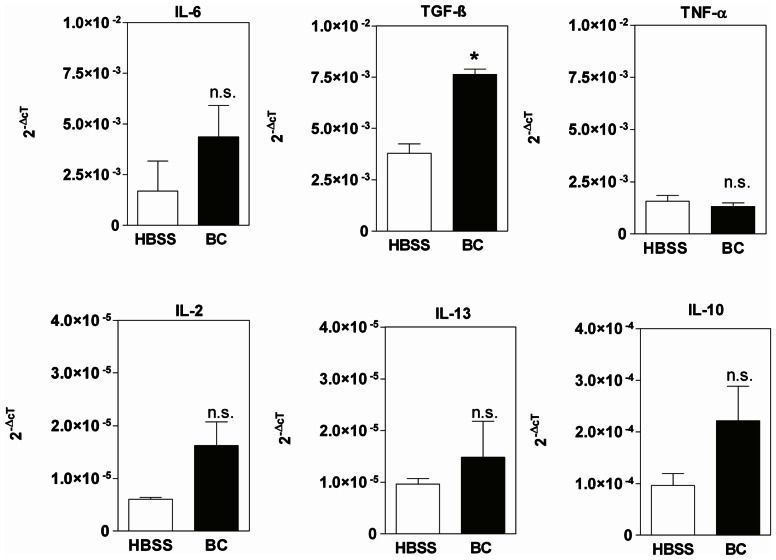
BC influences intestinal cytokine milieu. RNA-transcript levels of TGF-β but not IL- 6, 2, 13, 10, and TNF-α were significantly elevated in mouse proximal colon exposed to BC compared to Hank’s balanced salt solution control group (HBSS) *p<0.05, n = 4–6.

In order to explore effects of BC on the intestinal cytokine milieu, we performed an *in situ* perfusion experiment. Mice colons were *in situ* cannulated and perfused with 5% BC diluted in HBSS for 1 h. Cytokine expression was estimated in colonic tissue by real-time PCR analysis. As shown in Fig. 5, BC-exposure stimulated the intestinal expression of both, inflammatory and anti-inflammatory cytokines. There was a trend towards elevated IL-6 (p = 0.06) mRNA transcript levels after short-time exposure of mouse colon epithelium with BC compared to HBSS controls. The expression of other pro-inflammatory cytokines, which induce claudin-2 (TNF-α, IL-13) was not influenced by BC.

Moreover, short-time BC exposure induced significantly higher amounts of anti-inflammatory TGF-β (p = 0.03) and insignificantly elevated levels of IL-10 (p = 0.06) within the colonic epithelium compared to control. These data were of strong interest since TGF-β is well-known to down-regulate ion permeability by the induction of sealing TJ proteins [28] (Fig. 5).

## Discussion and Conclusions

In a previous study we demonstrated that prophylactic treatment with oral colostrum improves dextran sodium sulfate-induced murine colitis. Colostrum was found to induce regulatory immune cells and to accelerate epithelial restitution [16]. In the present study we therefore explored potential mechanisms that may mediate the observed effect of BC. Epithelial integrity is partly regulated by TJ modifications in response to various external stimuli [29], [30], and genetic variations of TJ genes affect epithelial barrier function [31]. In accordance with this, functional TJ abnormalities are associated with the pathogenesis of Crohn’s disease and ulcerative colitis [17], [27]. Thus, we first analyzed the effects of BC on the expression of intestinal TJ proteins in mouse intestine. The analyses revealed an enhanced claudin-2 protein expression, whereas the expression of other analyzed tightening (claudin-3, claudin-4) or pore-forming claudins (claudin-10, claudin-15) remained unaffected. Enhanced claudin-2 expression after BC-exposure was confirmed in differentiated human colonic epithelial cells.

Paradoxically, the increased expression of the channel-forming claudin-2 protein after BC incubation was not paralleled by the expected decrease but by an increase in TER. This effect was specific because exposure of differentiated, filter grown Caco-2 cells to IL-6 stimulated claudin-2 expression and resulted in the expected decrease in TER. Furthermore, the unchanged permeability of 4 kDa-FITC-Dx and the unaffected expression of IL-8 argue against an unspecific or toxic effect of BC on Caco-2 cells.

In addition, we found similar effects on claudin-2 expression and TER in Caco-2 cells after incubation with FBS, arguing for protein-specific effects of BC. Of note, in our previous studies prophylactic feeding with bovine serum albumin did not improve clinical or histological activity of colitis in mice and provoked toxic side effects [16]. Since both colostrum and FBS contain an undefined number of biological constituents [11], its mode of action might be probably multifunctional and its modulatory effects therefore not due to a single component. Beyond the different immunoglobulin subclasses (Table 1), colostrum comprises several factors that influence the proliferation and differentiation of epithelial cells. Biological active components of the innate defense such as soluble CD14 and Toll-like receptors in colostrum were shown to modulate immune responses against mucosal pathogens [32]. Colostral soluble CD14 neutralized bacterial antigens and induced differentiation of B cells [33], [34]. Oligosaccharides present in human and bovine milk influenced the composition of the intestinal microflora and prevented pathogen adhesion by the structural alteration of epithelial cell surface glycans [35]. In addition, colostral hormones, cytokines and growth factors influenced several cellular and metabolic functions of the intestinal epithelium. The immunomodulatory potential of milk-derived peptides and immunoglobulins in colostrum was demonstrated earlier [11], [36], [37]. However, previous data did not reveal beneficial effects of orally applied colostrum-derived peptides (lactoferrin) or immunoglobulins on epithelial restitution [38]. Furthermore, FBS has not been widely used as a control for BC; thus, further studies are required to analyze the comparability of BC and FBS.

Since intestinal homeostasis and TJ integrity are influenced by the cytokine milieu [2], and pro-inflammatory cytokines such as IL-6, TNF-α and IL-13 induce claudin-2 and decrease TER [21], we next analyzed effects of BC on the intestinal cytokine expression *in situ*. Pro-inflammatory cytokines such as IL-6, TNF-α and IL-13 are known to induce the channel-forming claudin-2 and to decrease TER [21]. We found TGF-β specifically induced by BC while intestinal TNF-α and IL-13 expression were not altered by BC. In our present experiments short-time BC-exposure only slightly increased IL-6 cytokine expression in the perfused mouse intestine and BC did not increase IL-6 secretion in human epithelial cell lines (data not shown). Moreover, intestinal TNF-α and IL-13 expression were not changed by BC in the perfused mouse intestine.

In previous studies, BC stimulated IL-2, interferon-γ, and IL-10 cytokine production in human PBMC [39] and had modulatory effects on activated PBMC [40], [41]. However, in our perfusion experiments, intestinal expression of IL-2 and IL-10 were marginal, but not statistically significant increased suggesting effects of BC on IL-2 and IL-10-production rather in peripheral blood monocytes than in the intestinal epithelium.

In contrast to the unchanged expression of IL-2, IL-6, IL-10, IL13, TNF-α, we found significantly up-regulated RNA expression of TGF-β after short-time BC-exposure. TGF-β is a predominantly anti-inflammatory cytokine which is well-known to enhance barrier function and consecutively to increase TER [28]. Thus, our data provide indirect evidence for complex effects of BC on barrier-forming proteins that might finally cause the beneficial effects of BC observed *in vivo*.

Controlling clinical signs of inflammation and thereby improving the quality of patient’s life is the main goal of IBD treatment. Currently, the therapeutic end-point in the treatment of IBD is the remission of symptoms but emerging evidence indicates that sub-clinical mucosal inflammation and the intake of non-steroidal anti-inflammatory drugs that disrupt the barrier could lead to disease relapse [42], [43]. In a previous study, colostrum was able to prevent injury of the rat small intestine caused by non-steroidal anti-inflammatory drugs [14]. The present study gives evidence for colostrum as a prophylactic agent with properties that strengthen barrier function and favour epithelial restitution.
